# Resilience and adjustment trajectories amongst children in displacement-affected communities in Zarqa, Jordan

**DOI:** 10.29392/001c.18233

**Published:** 2021-01-05

**Authors:** Sabrina Hermosilla, Janna Metzler, Kevin Savage, Alastair Ager

**Affiliations:** 1 Institute for Social Research, University of Michigan, Ann Arbor, Michigan, USA; 2 Department of Population and Family Health, Mailman School of Public Health, Columbia University, New York City, New York, USA; 3 Humanitarian and Emergency Affairs, World Vision International, Geneva, Switzerland; 4 Department of Population and Family Health, Mailman School of Public Health, Columbia University, New York City, New York, USA; Institute for Global Health and Development, Queen Margaret University, Edinburgh, Scotland, UK

**Keywords:** mental health, global health, program evaluation, refugee

## Abstract

**Background:**

The experiences of protracted conflict and displacement are clear threats to children’s developmental progress. Understanding the factors that shape the trajectories of children’s well-being and adjustment in such contexts is important for informing interventions.

**Methods:**

We collected data at three time points from a sample of Syrian refugee and Jordanian children (n=650) residing in Zarqa, Jordan who met eligibility criteria for humanitarian programming. We assessed primary outcomes of protection concerns, caregiver stress, mental health, and developmental assets at three time points: baseline (T1), three months later (T2), and fifteen months after baseline (T3).

**Results:**

Over the fifteen-month study period (T1-T3) child protection concerns and mental health symptoms improved, caregiver stress remained constant, and developmental assets deteriorated. School attendance was independently associated with improvements in protection concerns (*β* = −1.05, *P*=0.01), caregiver stress (*β* = −0.66, *P* =0.02), and developmental assets (*β* = 3.84, *P* =0.02). Concern over lost livelihoods significantly predicted higher protection concerns (*β* = 4.08, *P* <0.001) and caregiver stress (*β* = 2.32, *P* <0.001). Attending child-focused programming did not significantly impact primary outcomes.

**Conclusions:**

This study documents the capacity for adjustment and adaptation of children in the context of protracted displacement. The significant influences of attending school and concern over lost livelihoods on observed trajectories indicate the importance of addressing structural factors, such as education and employment, in supporting processes of resilience in these populations. Programmatic activities for children may secure valuable shorter-term impacts but here, as elsewhere, failed to impact outcomes longer-term.

Conflict and displacement are clear threats to children’s mental health and developmental progress. By the end of 2018, 70.8 million people were displaced by armed conflict and violence, more than half were under the age of 18.^1^ Children are particularly vulnerable during emergency situations.^2–4^ Humanitarian crises have become protracted, lasting decades; 78% of refugees in 2018 were in exile for over five years.^1^ Children forced to resettle across international borders are faced with shifts in family dynamics and are often exposed to separation, exploitation and forced labor, sexual violence and rape, physical abuse, challenges in navigating a new education system, and food insecurity.^5–7^ While at increased risk, the specific mental health and development trajectories (and thus corresponding needs) of children are still poorly understood.^8^

After eight years of conflict, these threats and increased vulnerability are dire for children affected by the Syrian crisis. Syrian refugees account for over a quarter of the world’s 20 million refugees, with nearly 2.4 million children displaced in the neighboring countries of Jordan, Lebanon, Turkey, Iraq, and Egypt.^1,9^ Jordan is host to the 10^th^ largest population of refugees in the world equating to well over half of a million people, half of which are children.^1,10,11^ As most refugees within Jordan have settled within local host communities,^9^ the challenges, instability, and resultant deleterious health and developmental effects may influence both Syrian children and their Jordanian hosts, however their specific mechanism and trajectories are currently unclear.^12^ Threats to the longer-term development trajectories of children in these contexts have elevated child mental health and development to a central research, programmatic, and policy priority.^5,13,14^ Navigating early-life stressors – and associated instability – that are characteristic of displacement-affected communities can have severe and lasting effects on children’s mental health, developmental progress, and wellbeing across the life-course.^5,15–17^ Experiencing social adversity during childhood is associated with longer-term developmental problems such as speech and language difficulties, learning and physical disorders, and emotional and behavioral problems.^12,18–20^ When these exposures are at a population level (e.g. displacement-affected communities), social adversities - and resultant deleterious outcomes – cluster, with the potential to impact communities, countries, and regions for a generation.^19,21–23^ As the Syrian conflict continues, the potential for an entire generation of children to be ‘lost’ in its wake has united diverse actors towards a coordinated regional initiative to ensure children have access to resources and essential interventions.^24^

The concept of resilience is a useful frame for children’s experience in situations of extreme adversity and is increasingly adopted by humanitarian practitioners and donors to understand and articulate the nature and role of service provision within the broader socio-ecological system of support, care, and protection critical to the adjustment and wellbeing of children.^25^ With this framework, mental health and psychosocial support interventions frequently seek to strengthen adaptive capacities and assets linked to healthy developmental milestones for children, as well as provide resource support to the child’s protective environment, comprised of their family, peers, school, and various social and institutional support structures.^25^ Resilience is thus not just the capacity of individuals to access critical resources that sustain wellbeing, but also the interaction and communication between groups and individuals at various socio-ecological levels.^26^ Utilizing resilience-based approaches provides a unique opportunity to examine the often complex and dynamic processes of resource acquisition and negotiation that occur during periods of displacement while simultaneously monitoring and holistically assessing these resource needs.^26,27^ As a nascent framework, the evidence-base for such interventions is lacking.

This study seeks to identify the factors that were predictive of better adjustment and adaption amongst displacement-affected children over an extended period. This included engagement in child-focused programming activities, schooling, etc., as well as broader demographic predictors. Understanding the factors that shape the trajectories of children’s well-being and adjustment in such contexts is essential in guiding future interventions.

## METHODS

### THE CURRENT STUDY

This prospective longitudinal study maps the trajectories of Syrian refugee and Jordanian children in a Jordanian community heavily affected by displacement resulting from the Syria crisis. The sampling frame constitutes children registered in 2014 for program activities provided by the Islamic Charity Center – in partnership with World Vision Jordan – in the southwest section of the urban capital of the Zarqa Governorate, Az-Zarqa. The study tracks protection concerns, caregiver stresses, mental health and psychosocial wellbeing, and development outcomes for these children over a fifteen-month period to identify which factors predicted more favorable outcomes.

Primary socio-demographic factors measured to discern their potential influence on outcomes include age, gender, nationality, school enrollment, and household composition, size, and livelihoods.

Another factor considered was engagement in the program activities offered by the Islamic Charity Center in the early months of refugee settlement in the area. Detailed intervention information is available elsewhere; briefly, this programming was delivered in the form of establishment of a Child Friendly Space (CFS).^28^ The CFS intervention was offered three days per week for two hours a day separately to two age ranges (5 to 12 years and 13 to 17 years) for up to 12 weeks. Children participating in the CFS were exposed to various structured activities, such as drama, singing, handicrafts, drawing, games, puzzles, storytelling, and informational videos. Children also engaged in sessions on life skills, such as hygiene, community mapping, and the importance of volunteerism.

### STUDY DESIGN

The study protocol was conducted with ethical approval from the Columbia University Medical Center (Reference AAAJ4352) and the Ministry of Social Development for the Hashemite Kingdom of Jordan. The research was conducted in collaboration with Columbia University Middle East Research Center, World Vision Jordan, the Islamic Charity Center, and the Family Guidance and Awareness Center. The study location was the first site with new programming of CFS during the research time period, as previously determined by the strategic priorities of implementing partners.

Survey instruments and consent were developed in English, adapted from Arabic-validated where available,^29^ then translated into Arabic by a native speaking content-specialist and back translated into English. Informed consent was obtained in Arabic from all adult participants. Caregivers were asked to participate if their child (aged six to seventeen) was registered for the intervention as either a participant or a waitlist control member. Caregivers were interviewed if their child was aged six to nine at the time of interview. For children aged ten to seventeen, after the study team documented their caregiver’s consent, children were approached and provided their assent and were interviewed directly. Outreach was conducted in the neighborhoods adjacent to the community center (populated by primarily lower income Jordanians; Syrian and some Iraqi and Palestinian refugees) in the week preceding intervention registration. Children aged six to seventeen were eligible for intervention programming within the center. All families arriving during the registration window were provided with written or oral consent forms related to the evaluation interviews and asked to voluntarily participate in baseline interviews.^28^ Upon completion of the registration week, children were allocated into age-disaggregated activity sessions (intervention) on a first come, first serve basis; children not included in the first round of programming were placed on a waitlist for subsequent rounds of programminfg (waitlist-control).

**Figure 1.**
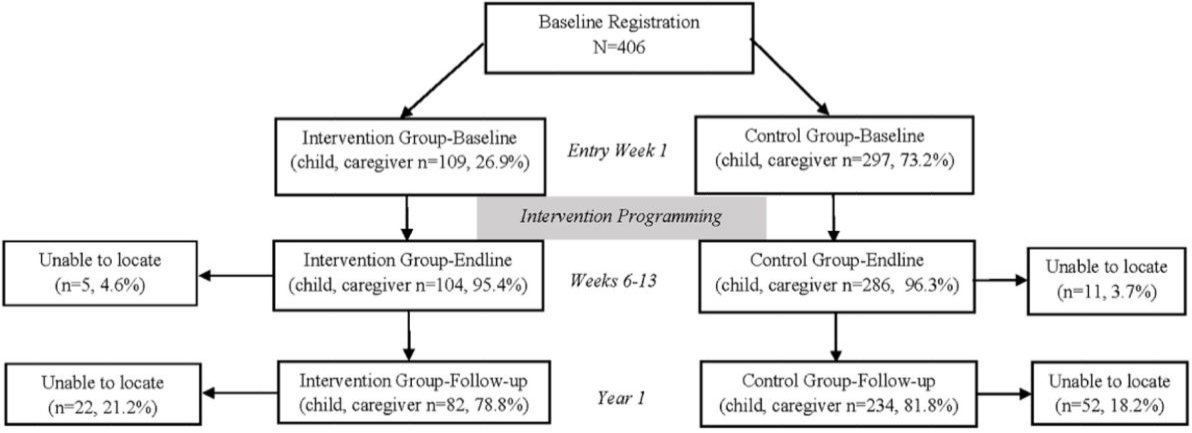
Study flow diagram .

Survey data collection took place in February 2014 (T1), June 2014 (T2), and from June to September 2015 (T3, initiating approximately one year after intervention completion, to estimate maintained long-term effects). The field team consisted of monitoring and evaluation specialists, graduate students, and a team of local enumerators. Interviews were conducted in semi-private locations in a large room within the community center at T1 and T2 and at a partner facility at T3. Participants were reimbursed for transportation at T3 in appreciation for their time, participation, and travel to the new partner facility, some distance from the original interview location (Figure 1).

### PRIMARY OUTCOMES

#### Protection concerns.

A question from the inter-agency Child Protection Rapid Assessment was adapted for use in Jordan^30^ to assess protection concerns. Children and caregivers were asked which, if any, of these concerns about child protection were a source of stress since coming to Jordan (or since the recent Syrian migration flow to Jordan) or any others: ‘not being able to go back to school’, ‘not being able to return home’, ‘losing their home and belongings, ‘being separated from their friends’, ‘being separated from their families (extended)’, ‘tension within the family (extended)’, ‘domestic violence’, ‘sexual violence’, ‘lack of jobs/inability to provide an income for family’, ‘nightmares or bad memories’, ‘crowdedness within your living area’, ‘lack of safe play areas’, ‘lack of hope for their future.’ Other child protection concerns were recorded as open-ended responses.

#### Caregiver stresses.

A further question from the interagency Child Protection Rapid Assessment was adapted for assessment of caregiver stresses in this context.^30^ Children and caregivers were asked which, if any, of these stresses had been a concern for caregivers since coming to Jordan or any others: ‘lack of food’, ‘not enough water’, ‘lack of shelter’, ‘lost property’, ‘lost livelihood’, ‘children’s safety’, ‘lack of education’, ‘decreased access to healthcare.’ Other sources of stress for caregivers were recorded as open-ended responses.

#### Mental health.

The 21-item Arab Youth Mental Health scale,^29^ a screening tool for depression and anxiety in youth, was used to assess mental health. It includes questions such as ‘During the last week I was bored and hated my life’ and ‘During the last week I was having a lot of headaches, stomach-aches, and nausea’. Items are rated on a scale: 1 = Rarely, 2 = Sometimes; 3 = Always, with higher scores indicative of more symptoms of anxiety and depression.

#### Developmental assets.

The 13-item Emergency Developmental Assets Profile (EmDAP)^31^ assessed development assets within eight categories (e.g. positive identity, constructive use of time, social competencies) through child-completed items such as ‘I feel optimistic about the future’ and ‘I think it is important to help people’. Items are rated on a scale: 0 = Rarely, 1 = Sometimes, 2 = Often, 3 = Almost Always, with higher scores indicative of a greater amount of assets essential for healthy developmental progress. Quartile ranges indicating *Good, Adequate, Vulnerable*, and *Highly Vulnerable* levels of developmental assets vary across cultures. A caregiver-reported developmental assets Caregiver Rating of Developmental Assets (CDRA) profile – mirroring items of the EmDAP – was administrated to caregivers to answer in relation to their perceptions of their child.

### ADDITIONAL MEASURES

#### Socio-demographic variables.

Individual items related to child age, gender, and nationality were asked at each time period (T1, T2, and T3). Household factors such as: vulnerability designation (developed by program team around standards for beneficiary reporting; included factors: greater than six in household, primary caregiver is only parent in household or has chronic disease, and child in household has disability or chronic disease), primary caregiver relation to children (e.g., biological mother, father, aunt, uncle, grandparent, etc.), nationality, household size, and primary livelihoods, were also collected at each time period (T1, T2, T3).^28^

#### Program activities.

Children and caregivers were asked to report frequency of attendance for the CFS program (T1 and T2); for formal schooling (T1, T2 and T3); and for extra-curricular activities (T1, T2, and T3). CFS self-reported attendance was cross-referenced with facility-kept records to determine item reliability.

### STATISTICAL ANALYSIS

After quantitative data were cleaned, univariate and bivariate analyses were conducted to describe the sample and explore key patterns by age group, gender, and CFS attendance status. Cronbach’s alpha and Kuder-Richardson Formula 20 (KR20) were run to explore scale reliability by age and gender subgroups. Histograms and boxplots were created to explore quartile distribution of key outcome variables and normality distribution assumptions. Longitudinal generalized linear multivariable (GLM) models, controlling for design effects, were estimated based on important (*P*<0.10) bivariate findings and variables of epidemiologic significance, by outcome and age group, to explore unique contribution of key factors to main outcomes. Model diagnostics, including testing for multicollinearity (through variance inflation factors) amongst final model variables, were completed on all GLM models. To estimate program effect, we calculated crude Cohen’s d for each study outcome (recoded when necessary so that a positive value indicates a salubrious effect). A Cohen’s d of 0.20 is considered a small effect, 0.50 a moderate effect, and 0.80 or above a large effect.^32^ Observations with missing data were pairwise deleted. All analyses were conducted in STATA 14.2 (StataCorp, College Station, TX).

## RESULTS

### CHARACTERISTICS OF THE SAMPLE

235 caregivers of young children aged 6 to 9 years were traced over time with 168 retained for data collection at T2 and 141 retained at T3. 179 children aged 10 to 12 years were traced throughout the study period with 120 retained at T2 and 108 retained at T3. 236 older children aged 13 to 18 were traced throughout the study period with 132 retained at T2 and 132 retained at T3 (see Figure 1). 99.6% of the sample was missing two or fewer analytic variables. The sample was comprised of 59.4% Syrian children, 34.2% Jordanian children, and 5.9% Palestinian children (see Table 1). Children who reported at T2 to have attended CFS regularly (reported ‘sometimes’ or ‘always’ attended) were designated for analysis as ‘attenders’ (109 at T2, 72 at T3). Those who, despite registration at T1, reported ‘never’ attending at T2 were designated ‘non-attenders’ (297 at T2, 193 at T3). There were no significant differences between CFS attender and non-attender groups on baseline outcome measures or by composition, including sex (*χ*^2^=0.19, *P*=0.66), nationality (*χ*^2^=0.01, *P*=0.93), vulnerability designation (*χ*^2^=0.55, P=0.46), or formal school attendance at T1 (*χ*^2^=1.51, *P*=0.22) except for developmental assets where CFS attenders had slightly higher baseline levels as compared to those who did not attend (*P*=0.03). After primary CFS activities were concluded (between T2 and T3), approximately 59.1% (n=114) of the original CFS non-attenders (waitlist control) were offered and participated in programming. This subgroup was removed from further T2-T3 analyses related to CFS attendance efficacy such that ‘non-attenders’ are defined as never having attended CFS programming.

Bivariate findings offered additional exploration into factors that may be associated with the main longitudinal outcomes. After adjusting for age, gender, nationality, baseline vulnerability, school participation, and T1 and T2 outcome scores, results from longitudinal GLM models are reported in Table 2 for one-year predictors of outcome scores and Table 3 for impact of CFS attendance at T2. Intervention attendance rarely predicted primary program outcomes and no Cohen’s d estimates were statistically significant (*P*<0.05, Table 3).

#### PROTECTION CONCERNS

**Figure 2.**
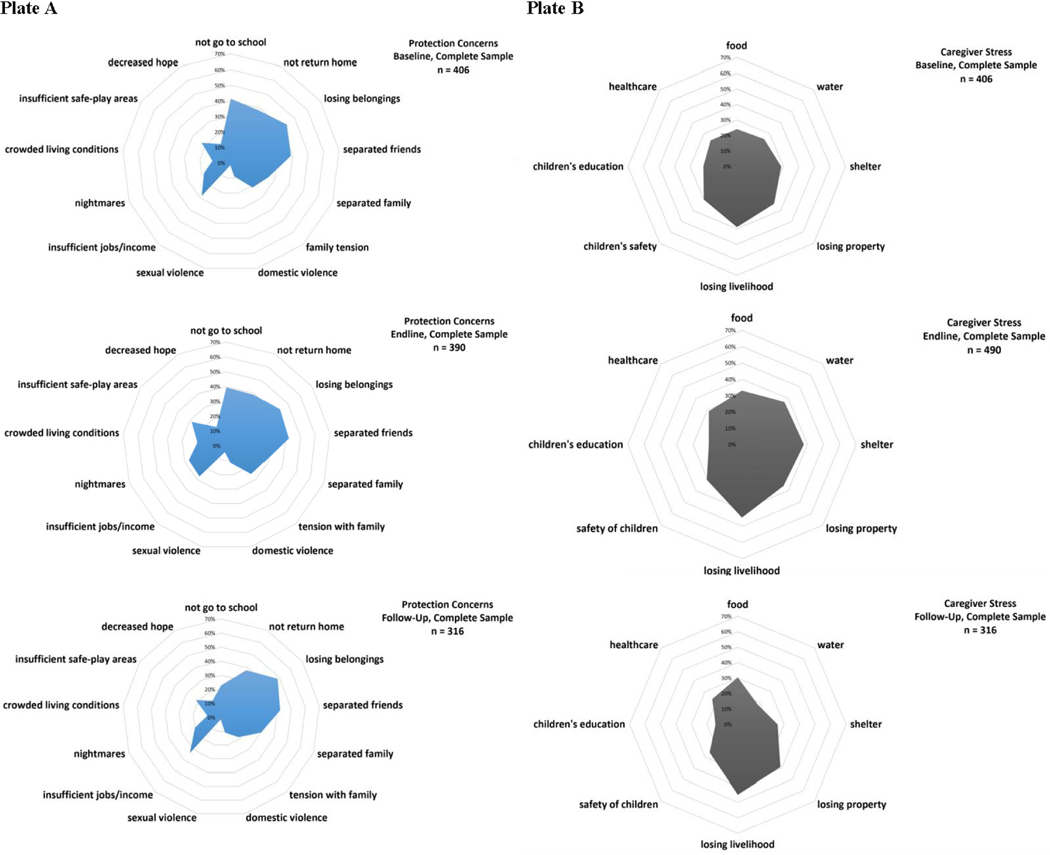
Spider diagrams. A. Protection concerns. B. Caregiver stresses.

The type of reported child protection concerns did not change appreciably over time (Figure 2, Plate A), except that by follow-up (T3), school attendance ceased to be a highly reported concern. At the end of the intervention (T2) fewer concerns were reported across the entire sample, as compared to T1, but showed no statistically significant changes within age-group stratified analyses (data not shown). While children aged 10–12 who attended CFS reported fewer concerns than those who did not attend (*β*=−1.16, *P*=0.05, Table 3) immediately after the intervention (T2), CFS attendance, gender, and vulnerability were not predictors of protection concerns at T3 (Table 2). Across all age ranges at T3, those who reported caregiver stress related to lost livelihood and being of non-Jordanian nationality (Syrian or Palestinian) reported increased protection concerns (nationality: all ages *β* =−1.65, *P*<0.001; 6–9 y. *β* =−2.74, *P*<0.001; 10–12 y. *β* =−1.66, *P*<0.001; 13–18 y. *β* =−0.99, *P*=0.06; lost livelihoods: all ages *β* =4.08, *P*<0.001; 6–9 y. *β* =3.42, *P*<0.001; 10–12 y. *β* =4.20, *P*<0.001; 13–18 y. *β* =4.55, *P*<0.001: Table 2). Children who attended school at both baseline (T1) and follow-up (T3) reported lower levels of protection concerns at follow-up (T3) than those who did not attend school. However, those who attended school at endline (T2) reported more protection concerns at follow-up (T3) than those who did not attend, especially amongst younger (6–9 year old) children (Table 2 and Online Supplementary Document, Table S1).

#### CAREGIVER STRESS

Caregiver stresses remained similar throughout the study period, with the exception that at T3 water had ceased to be a highly reported source of stress (Figure 2, Plate B). CFS attendance, gender, and vulnerability were not predictors of more caregiver stress sources (Table 2). Jordanian nationals reported fewer sources of caregiver stress at followup (T3), as compared to non-Jordanian nationals, across all age groups (all ages *β* = −2.06, *P*<0.001; 6–9 y. *β* =−2.65, *P*<0.001; 10–12 y. *β* =−1.62, *P*<0.001; 13–18 y. *β* =−1.76, *P*<0.001, Table 2). Those who attended school at baseline (T1) reported fewer caregiver stresses at follow-up (T3) (all ages *β* =−0.66, *P*=0.02; 6–9 y. *β* =−0.75, *P*=0.11; 10–12 y. *β* =−0.18, *P*=0.78; 13–18 y. *β* =−0.8, *P*=0.32, Table 2).

#### MENTAL HEALTH

**Figure 3.**
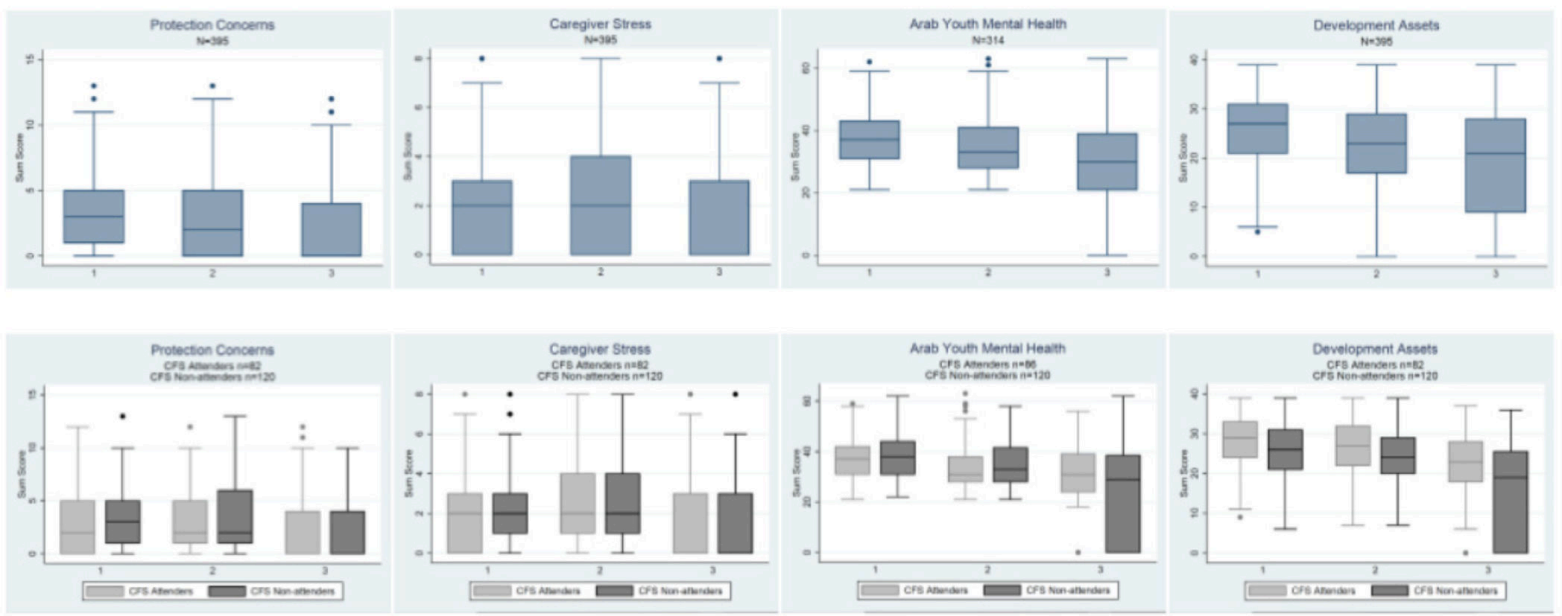
Trends in protection concerns, caregiver stress, mental health symptoms, and developmental assets over time for all children and by intervention attendance. Notes. Trends across time for Arab Youth Mental Health - in both complete sample and in sample stratified by attendance – were statistically significant (*P*<0.05). Time periods 1, 2, and 3 correspond to baseline, endline (three months post-baseline, intervention end), and follow-up (one year post-baseline).

Overall, mental health symptoms consistent with the Diagnostic and Statistical Manual of Mental Disorders - IV criteria for depression and anxiety remained below clinical levels and decreased across the study period (Figure 3). Amongst older children only, boys reported fewer symptoms than girls of the same age (T3) (13–18 y. *β* =−5.98, *P*=0.003, Table 2). Only Jordanian 10 to 12 year old children reported fewer symptoms as compared to non-Jordanian nationals (10–12 y. *β* =−4.63, p=0.03, Table 2). Older children reporting the stress related to lost livelihoods at follow-up (T3) reported lower levels of symptoms (13–18 y. *β* =−6.97, *P*<0.001, Table 2) as compared to those reporting higher stress related to lost livelihoods. CFS attendance, vulnerability, and school attendance were not predictors of mental health symptoms over time.

#### DEVELOPMENTAL ASSETS

Overall, developmental assets declined across the study period (Figure 3). Vulnerability (except for younger children), gender, and nationality were not significant predictors of developmental assets over time. Amongst young children, those who reported higher vulnerability at follow-up (T3) reported lower levels of developmental assets at follow-up (T3) (6–9 y. vulnerability: *β* =−6.84, *P* =0.02; Table 2). Young children who had higher levels of depression and anxiety at follow-up (T3) also reported slightly higher developmental assets at follow-up (T3) (6–9 y. *β* =0.52, *P*<0.001). Children aged 10 to 12 years attending school at endline (T2) reported, on average, more developmental assets than those children of the same age not attending school (10–12 y. *β* =9.07, *P*=0.002, Table 2). Children who attended CFS reported more assets at baseline (T1) and endline (T2) than those who did not attend (*β*=1.69, *P*=0.02, Table 3); however, this finding did not hold through age subgroup analyses, or at follow-up (T3).

#### NATIONALITY

We explored the role of nationality on primary study outcomes across all analyses (Online Supplementary Document, Table S3). Jordanian children, as compared to non-Jordanian children, had better baseline levels of primary outcomes (protection concerns, caregiver stress, mental health, and developmental assets). In all subsequent analyses, Jordanian children, as compared to non-Jordanian children, continued to do better on most outcomes (Tables 2, S1, S2, available online). Jordanian children, however, were not more likely to participate in the intervention than nonJordanian children (Online Supplementary Document, Table S3).

#### SCHOOL ATTENDANCE

We conducted additional sub-analyses into the impact of school attendance on primary outcomes. Over the study period school attendance increased, with 75.9% (n=308) attending at baseline (T1), 77.6% (n=326) at endline (T2, nonstatistically significant change), and 86.7% (n=261) at follow-up (T3, P<0.001 change, data not shown). There were no statistically significant differences in school attendance between those who attended CFS and those who did not – both across the entire sample and within subgroup age analyses (Table 1, S4, available online) – nor did school attendance predict CFS attendance. Those who attended school at baseline (T1) reported fewer child protection concerns (complete *β* =−1.33, *P*=0.02; 6–9 y. *β* =−2.71, *P*=0.01, Online Supplementary Document, Table S1) and caregiver stress (complete *β* =−1.37, *P*=0.002; 6–9 y. *β* =−2.50, *P*=0.003, Online Supplementary Document, Table S1) at follow-up (T3) than those who did not. Those who attended school at follow-up (T3) reported higher developmental assets (complete *β* =5.08, *P*<0.001; 13–17 y. *β* =5.56, *P*<0.001, Online Supplementary Document, Table S1) at follow-up (T3) as compared to those who did not attend school at follow-up (T3). As in other models, Jordanian children, as compared to non-Jordanian children, generally had better outcomes at follow-up (T3) (protection concerns complete *β* =−3.26, *P*<0.001; caregiver stress complete *β* =−2.55, *P<*0.001; mental health complete *β* =−3.80, *P*=0.005; developmental assets complete *β* =2.08, *P*=0.008; Online Supplementary Document, Table S1). Models exploring participation in extracurricular activities, excluding the primary intervention, did not produce statistically significant results nor change the overall effect of schooling on primary outcomes (data not shown).

#### LIVELIHOOD LOSS STRESS

Given the emerging importance of stress related to livelihood loss in predicting the primary study outcomes, we conducted additional sub-analyses to better understand its predictive role on primary outcomes. Reported stress related to loss of livelihood or fear of livelihood loss was the most often endorsed source of caregiver stress (Figure 2, Plate B), remained constant across the study period (T1: 27.7%; T2: 27.4%; T3: 33.2%), did not differ between intervention attenders and non-attenders at baseline (T1, Online Supplementary Document, Table S4), and did not predict intervention attendance. Those who endorsed stress related to livelihood loss (or fear of livelihood loss) at follow-up (T3) had higher levels of child protection concerns (complete β =4.60, *P*<0.001; 6–9 y. *β* =4.54, *P*<0.001; 10–12 y. *β* =4.61, *P*<0.001; 13–17 y. *β* =4.69, *P*<0.001, Online Supplementary Document, Table S2). Stress related to livelihood loss (or fear of loss) did not significantly predict developmental assets or mental health symptoms over the study period, save for older children (13–17 y. mental health: T1 stress *β* =7.09, *P*=0.002, T3 stress *β* =−6.97, *P*=0.007; developmental assets: T2 stress *β* =−3.72, *P*=0.03, Online Supplementary Document, Table S2). Again, in these models, Jordanian children, as compared to non-Jordanian children, generally had better outcomes at follow-up (T3) (protection concerns complete *β* =−1.74, *P*<0.001; caregiver stress complete *β* =−3.05, *P=*0.03; developmental assets complete *β* =2.55, *P*=0.004; Online Supplementary Document, Table S2).

## DISCUSSION

This study found that over the fifteen-month study period, amongst displacement affected children in Jordan, overall child protection concerns and mental health symptoms improved, caregiver stress remained statistically unchanged, and developmental assets deteriorated. Attending child-focused programming in Zarqa, Jordan in the period between T1 and T2 did not significantly impact child protection concerns, caregiver stress, or developmental assets at T2 or T3 overall. In contrast, attending school and concerns over lost livelihoods had significant influence on primary study outcomes. Similar long-term mental health trajectories – beyond intervention effects – towards symptom alleviation have been documented in other intervention studies: among Syrian refugee and Jordanian youth,^13^ war-affected youth in Sierra Leone,^33^ and political violence-affected youth in Indonesia.^34^ Observational studies have also documented symptom alleviation overtime,^35,36^ even in formal symptom class trajectory analyses (beyond scope of current study).^37^

Declining developmental assets over the study period underscore the uncertainty faced by displaced and local children and adolescents in Jordan. Unfamiliar surroundings, fractured communal and social support linkages, and poor economic prospects have strained the protective environment for children affected and displaced by the Syrian conflict.^38^ While not statistically significant across all age groups, high familial vulnerability and caregiver stress predicted lower - and school attendance predicted higher - developmental assets (Table 2), consistent with an understanding of familial and community resources as key assets for development. ^39^ The overall trajectory for participants of improved mental health but diminished development assets indicates that resilient adjustment may not so much be driven by – but come through the investment and expenditure of – personal and community assets. ^27^

Schooling plays an essential role in both promoting resilience and providing a safe environment where children and adolescents are potentially protected from additional potentially traumatic events and stressors. As compared to other samples of displaced Syrian youth in Jordan where approximately half of surveyed individuals could not continue their education,^40,41^ over 75% of our sample attended school during the study period. Attending school predicted improvements in child protection concerns, caregiver stress, and higher levels of developmental assets (Table S1, available online). Similar protective influences of schooling have been documented among former child soldiers^42,43^ and in low-income contexts outside humanitarian settings.^44^ However, perhaps because of the potential benefits of attending school, pre-adolescent and adolescent children sometimes face rising concerns with heightened awareness of the challenges of attending a new school. School is not immune from the challenges that cut across existing child protection systems. While not documented in this analysis, Syrian children wishing to enroll in school face many of the same barriers as do low-income youth and caregivers looking to access existing child protection systems, with barriers ranging from child labor and exploitation to precarious local conditions.^45^

Economic stability plays an important role in community, family, and individual security. We found that stress associated with livelihood loss, or the fear of livelihood loss, was associated with increased child protection fears and caregiver stress. As the conflict in Syria rages on, displaced children are confronted daily with the realities of living a life in exile where their developmental trajectories and economic opportunity remain uncertain. The fracturing of communal and social mechanisms of support and protection, such as family and friends, have contributed to rising concerns about the safety of children within the displaced environment and the stress response of caregivers with poor employment opportunities. External programming can either support or undermine not only the uncertainty and stress of livelihood securement, but also the complex communal resources and protective mechanisms.^23^

This study suggests that attendance at the programming in Zarqa, Jordan, did not, overall, improve child or adolescent outcomes in the short term. The inability to identify an intervention impact could reflect a lack of a true impact, consistent a recent meta-analysis of psychosocial interventions.^46^ Amongst older (13–17) children, however, boys who attended the intervention reported fewer symptoms of depression and anxiety (*β* =−5.90, *P*=0.003), as compared to girls, over time. This is consistent with existing literature on impact evaluations that documents heterogeneous impact across participant subgroups, such as age, gender, socioeconomic status, and access to education.^47–49^ Given that girls and boys had similar levels of mental health symptomology and attendance at baseline, the relative improvement of boys, especially older boys, over girls, indicates a gendered programmatic effect.

This study provides meaningful insight into the trajectory of children and adolescents in Jordan. However, there are certain limitations to this study. The study only evaluated one CFS intervention site and was based on a non-randomized assignment to intervention and control. A high percentage of individuals reported having never participated in other non-CFS or school activities (T1: 86.0%; T2: 74.1%; T3: 88.0%). Given the importance of other activities^50^ for child and adolescent development, it is important to consider this population makeup when comparing to other program evaluations. Additionally, for one of our primary outcomes, mental health, we had high item-level missingness (35%). However, item-level missingness analyses detected no patterning to or measured predictors of the missingness. As with all self-report (or caregiver report) studies, issues of social desirability could lead to underreporting of some conditions deemed stigmatized or unacceptable in this community. Every effort was made during the design and implementation of the study to ensure participants felt comfortable and secure in responding to items, including using trained data collectors experienced in sensitive data collection. This study explores children and adolescents currently residing in Jordan, additional information on study participants before displacement, including more accurate data on displacement timing, events would enable a more complete understanding of the impact of displacement on this population,

In conclusion, while programme benefits were marginal, the relatively significant influences of attending school and concerns over lost livelihoods among the study population suggests that to improve child and adolescent wellbeing in contexts of displacement, advancements in access to structural determinants of health, such as education and employment, are essential.

## Supplementary Material

supplemental tables

1

## Figures and Tables

**Table 1.** Baseline participant demographics and descriptive characteristics by age

	Entire sample		Children 6–9		Children 10–12		Children 13–17	
	(n = 406)		(n = 165)		(n = 114)		(n = 127)	
**Age (standard deviation)**	10.7	3.1	7.6	1.1	11	0.9	14.4	1.3
**Gender (%)**								
Female	220	54.2%	84	50.9%	59	51.8%	77	60.6%
**Nationality (%)**								
Jordanian	139	34.2%	50	30.3%	48	42.1%	41	32.3%
Syrian	241	59.4%	110	66.7%	62	54.4%	69	54.3%
Palestinian	24	5.9%	5	3.0%	5	4.4%	14	11.0%
**Vulnerability designation (%)**								
Yes	49	12.1%	27	16.4%	8	7.0%	14	11.0%
**Formal school attendance (%)**								
Yes	308	75.9%	129	78.2%	95	83.3%	84	66.1%
**Extracurricular activities (%)**								
Always	19	4.7%	6	3.7%	5	4.4%	8	6.3%
Sometimes	38	9.4%	9	5.5%	14	12.3%	15	11.8%
Never	348	85.9%	149	90.9%	95	83.3%	104	81.9%
**Primary study Measures (standard deviation)**								
Protection concerns	3.1	2.9	3.7	3.3	2.4	2.4	2.9	2.8
Caregiver stress	2.2	2.1	2.6	2.2	1.8	1.9	1.9	2.0
Mental health	37.7	8.6	38.9	8.8	35.5	6.3	38.1	8.8
Development assets	26.5	6.8	25.5	6.4	27.6	6.2	25.5	8.2

**Table 2.** Primary one-year predictors of child protection concerns, caregiver stress, mental health, and developmental assets by age

	Complete Sample	Children 6–9	Children 10–12	Children 13–17
	β (95% CI)	β (95% CI)	β (95% CI)	β (95% CI)
**Protection concerns**	n=277	n=117	n=81	n=79
Age	−0.01 (−0.12–0.05)	−0.02 (−0.41–0.37)	**0.67 (0.18–1.15)** **	0.14 (−0.20–0.48)
Gender, (ref: girls)	0.01 (−0.48–0.49)	−0.31 (−1.10–0.48)	0.72 (−0.17–1.61)	0.20 (−0.67–1.07)
Nationality (ref: non-Jordanian)	−1.65 (−2.29– –1.02)***	−2.74 (−4.04– –1.44)***	−1.66 (−2.64– –0.69)***	−0.99 (−2.04–0.05)
Attended school, baseline	−1.05 (−1.87– –0.23)*	−1.68 (−3.17– –0.18)*	−	−
Attended school, follow-up	−0.90 (−1.79– –0.01)*	−	−	−
Lost livelihood, follow-up	**4.08 (3.49–4.67)** ***	**3.42 (2.41–4.43)** ***	**4.20 (3.11–5.28)** ***	**4.55 (3.41–5.70)** ***
**Caregiver stress**	n=328	n=168	n=81	n=79
Age	−0.06 (−0.14–0.02)	−0.07 (−0.40–0.27)	0.49 (0.02–0.96)*	0.06 (−0.25–0.38)
Gender, (ref: girls)	−0.06 (−0.51–0.40)	−0.38 (−1.09–0.34)	0.37 (−0.49–1.22)	0.06 (−0.77–0.83)
Nationality (ref: non-Jordanian)	−2.06 (−2.61– –1.50)***	−2.65 (−3.59– –1.71)***	−1.62 (−2.51– –0.72)***	−1.76 (−2.68– –0.83)***
Attended school, baseline	−0.66 (−1.22– –0.09)*	−0.75 (−1.67– –0.17)	−0.18 (−1.44–1.08)	−0.48 (−1.43–0.46)
**Mental mealth**	n=201	n=92	n=58	n=51
Age	**1.23 (0.59–1.87)** ***	−0.79 (−4.06–2.48)	−1.17 (−3.52–1.18)	−0.01 (−1.66–1.63)
Gender, (ref: girls)	−2.22 (−6.01–1.56)	0.29 (−6.19–9.81)	−1.05 (−5.31–3.20)	−5.98 (−9.96– –2.01)**
Nationality (ref: non-Jordanian)	−0.70 (−4.77–3.38)	−1.81 (−6.19–9.81)	−4.63 (−8.90– –0.35)*	−**2.78 (−7.29– 1.73)**
Lost livelihood, follow-up	−	−	−	−7.59 (−12.75– –2.44)**
**Developmental assets**	n=158	n=92	n=66	n=49
Age	0.20 (−0.18–0.57)	−0.56 (−1.85–0.74)	0.90 (−0.45–2.26)	0.29 (−0.87–1.45)
Gender, (ref: girls)	−1.05 (−2.09–0.81)	−0.37 (−2.61–3.34)	−0.79 (−3.29–1.70)	−1.20 (−4.19–1.80)
Nationality (ref: non-Jordanian)	1.43 (−0.70–3.56)	3.35 (−0.10–6.81)	0.43 (−2.04– 2.91)	−0.99 (−4.57–2.59)
Vulnerable, follow-up	−	−6.84 (−12.61 – –1.07)*	−	−
Attended school, endline	−0.50 (−3.58–2.59)	−	**9.07 (3.38–14.75)** **	0.71 (−2.87–4.30)
Attended school, follow-up	**3.84 (0.65–7.02)** *	−	−2.70 (−8.81–3.41)	−0.54 (−4.55–3.47)
Mental Health, follow-up	−	**0.52 (0.43–0.61)** ***	−	−
Caregiver stress, baseline	−0.83 (−1.49– –0.18)*	−0.71 (−1.43– –0.02)	−	−

Notes:

* <0.05;

** <0.01;

*** P<0.001.

Bold type indicates statistical significance P<0.05. – indicates variable was not included in final adjusted model.

**Table 3.** Four-month impact of intervention attendance on protection concerns, caregiver stress, mental health, and developmental assets by age

	Complete sample		Children 6–9		Children 10–12		Children 13–17	
	β (95% CI)	Cohen’s *d*	β (95% CI)	Cohen’s *d*	β (95% CI)	Cohen’s *d*	β (95% CI)	Cohen’s *d*
Protection concerns	−0.48 (−1.13–0.17)	0.09	−0.72 (−1.83–0.38)	0.24	−**1.16 (−2.32– 0.00)**	0.27	0.23 (−0.95–1.40)	−0.19
Caregiver stress	−0.19 (−0.62–0.25)	0.00	−0.26 (−1.01–0.50)	0.04	−0.66 (−1.46–0.14)	0.20	0.25 (−0.49–0.98)	−0.18
Mental health	−1.36 (−3.48–0.77)	0.18	−3.37 (−7.10–0.36)	0.48	−2.16 (−5.57–1.26)	0.20	1.88 (−2.09–5.86)	−0.12
Development assets	**1.69 (0.24–3.14)**	0.07	1.87 (−0.77–4.50)	0.03	0.78 (−1.76–3.33)	0.14	1.12 (−1.31–3.54)	0.05

Notes: Bold type indicates statistical significance P<0.05. No Cohen’s d were statistically significant. All models adjusted for subject’s age, gender, nationality, vulnerability status, school attendance, extra-curricular activities, caregiver stress, and baseline outcome values.
