# Supplementary material for: Resilience and adjustment trajectories amongst children in displacement-affected communities in Zarqa, Jordan
Source: J Glob Health Rep. Author manuscript; Available in PMC 2022 Feb 3. (PMC8813053; doi:10.29392/001c.18233)
Supplement: supplemental tables [file NIHMS1760452-supplement-supplemental_tables.docx]

**Supplemental Table 1:** School Attendance Association with Primary One-year Outcomes: Child Protection Concerns, Caregiver Stress, Mental Health, and Developmental Assets by Age

|  | **Complete Sample** |  | **Children 6-9** |  | **Children 10-12** |  | **Children 13-17** |
| --- | --- | --- | --- | --- | --- | --- | --- |
|  | **β (95% CI)** |  | **β (95% CI)** |  | **β (95% CI)** |  | **β (95% CI)** |
|  |  |  |  |  |  |  |  |
| **Protection Concerns** | **n=277** |  | **n=117** |  | **n=81** |  | **n=79** |
| Age | **-0.17 (-0.28- -0.06)**** |  | -0.02 (-0.47-0.43) |  | 0.50 (-0.18-1.17) |  | 0.28 (-0.19-0.75) |
| Gender, (ref: girls) | -0.06 (-0.69-0.57) |  | -0.10 (-1.02-0.82) |  | 0.49 (-0.69-1.66) |  | -0.09 (-1.32-1.14) |
| Nationality (ref: non-Jordanian) | **-3.26 (-4.01- -2.50)***** |  | **-4.92 (-6.18- -3.66)***** |  | **-2.53 (-3.84- -1.22)***** |  | **-2.03 (-3.44- -0.61)**** |
| Attended school, baseline | -0.75 (-1.79-0.29) |  | -1.22 (-2.95-0.50) |  | -0.52 (-2.51-1.47) |  | -0.50 (-2.39-1.39) |
| Attended school, endline | 1.08 (-0.10-2.25) |  | **3.15 (1.09-5.20)**** |  | 0.60 (-1.49-2.70) |  | -0.13 (-2.15-1.89) |
| Attended school, follow-up | -0.44 (-1.59-0.71) |  | -2.32 (-4.61- -0.03) |  | -0.30 (-2.56-1.96) |  | 0.35 (-1.34-2.03) |
|  |  |  |  |  |  |  |  |
| **Caregiver Stress** | 277 |  | 117 |  | 81 |  | 79 |
| Age | **-0.19 (-0.27- -0.11)***** |  | -0.10 (-0.43-0.23) |  | 0.45 (-0.03-0.93) |  | 0.06 (-0.26-0.39) |
| Gender, (ref: girls) | -0.11 (-0.56-0.35) |  | -0.30 (-0.99-0.39) |  | 0.40 (-0.44-1.25) |  | 0.08 (-0.79-0.95) |
| Nationality (ref: non-Jordanian) | **-2.55 (-3.08- -2.01)***** |  | **-3.97 (-4.88- -3.07)***** |  | **-1.67 (-2.56- -0.77)***** |  | **-1.74 (-2.69- -0.78)***** |
| Attended school, baseline | **-0.86 (-1.62- -0.10)*** |  | **-1.36 (-2.64- -0.08)*** |  | -0.30 (-1.72-1.12) |  | -0.37 (-1.72-0.97) |
| Attended school, endline | 0.66 (-0.19-1.52) |  | **1.60 (0.06-3.13)*** |  | 0.93 (-0.59-2.44) |  | -0.10 (-1.51-1.32) |
| Attended school, follow-up | -0.62 (-1.45-0.21) |  | -1.57 (-3.28-0.13) |  | -0.92 (-2.53-0.70) |  | -0.06 (-1.23-1.10) |
|  |  |  |  |  |  |  |  |
| **Mental Health** | 176 |  | 67 |  | 58 |  | 51 |
| Age | -0.22 (-0.66-0.21) |  | -0.52 (-2.64-1.59) |  | -0.55 (-2.98-1.89) |  | -0.31 (-2.15-1.54) |
| Gender, (ref: girls) | -1.94 (-4.37-0.50) |  | -0.15 (-4.43-4.13) |  | -0.19 (-4.57-4.19) |  | -6.65 (-11.25- -2.04) |
| Nationality (ref: non-Jordanian) | **-3.80 (-6.47- -1.14)**** |  | -4.12 (-8.87-0.64) |  | **-5.41 (-9.86- -0.96)*** |  | -3.00 (-8.34-2.35) |
| Attended school, baseline | 0.21 (-3.96-4.38) |  | 4.95 (-4.92-14.82) |  | 0.28 (-6.57-7.12) |  | -1.93 (-9.07-5.22) |
| Attended school, endline | 1.06 (-3.65-5.76) |  | -0.47 (-12.43-11.49) |  | 0.77 (-7.53-9.07) |  | 3.25 (-4.05-10.54) |
| Attended school, follow-up | 0.38 (-3.77-4.53) |  | -5.32 (-18.39-7.75) |  | 5.55 (-2.09-13.19) |  | -1.67 (-7.55-4.20) |
|  |  |  |  |  |  |  |  |
| **Developmental Assets** | 251 |  | 117 |  | 69 |  | 65 |
| Age | 0.21 (-0.03-0.45) |  | -0.07 (-0.98-0.84) |  | 1.07 (-0.34-2.49) |  | 0.48 (-0.74-1.71) |
| Gender, (ref: girls) | -0.33 (-1.74-1.07) |  | 0.37 (-1.60-2.35) |  | -2.00 (-4.45-0.45) |  | -0.69 (-4.21-2.83) |
| Nationality (ref: non-Jordanian) | **2.08 (0.55-3.61)**** |  | **3.84 (1.53-6.16)**** |  | 0.81 (-1.77-3.39) |  | -1.63 (-5.21-1.95) |
| Attended school, baseline | -0.84 (-3.18-1.50) |  | -0.75 (-4.37-2.88) |  | 2.34 (-2.22-6.89) |  | -2.43 (-7.63-2.77) |
| Attended school, endline | 0.29 (-2.42-3.00) |  | -1.88 (-6.24-2.49) |  | **6.97 (1.84-12.11)**** |  | 1.79 (-3.67-7.25) |
| Attended school, follow-up | **4.77 (2.13-7.42)***** |  | 3.84 (-1.14-8.83) |  | 2.40 (-2.62-7.43) |  | **6.00 (1.41-10.59)*** |
|  |  |  |  |  |  |  |  |

*Notes: *<0.05; **<0.01; ***P<0.001 All models adjusted for subject's age, gender, nationality, school attendance, extracurricular activities, caregiver stress, and baseline outcome values.*

**Supplemental Table 2:** Livelihood Loss Stress Association with Primary One-year Outcomes: Child Protection Concerns, Mental Health, and Developmental Assets by Age

|  |  | **Complete Sample** |  | **Children 6-9** |  | **Children 10-12** |  | **Children 13-17** |
| --- | --- | --- | --- | --- | --- | --- | --- | --- |
|  |  | **β (95% CI)** |  | **β (95% CI)** |  | **β (95% CI)** |  | **β (95% CI)** |
|  |  |  |  |  |  |  |  |  |
| **Protection Concerns** |  | n=277 |  | n=117 |  | n=81 |  | n=79 |
| Age |  | -0.04 (-0.12-0.05) |  | -0.02 (-0.41-0.36) |  | **0.70 (0.21-1.18)**** |  | 0.19 (-0.15-0.52) |
| Gender, (ref: girls) |  | 0.08 (-0.42-0.57) |  | -0.34 (-1.17-0.49) |  | 0.65 (-0.23-1.53) |  | 0.23 (-0.62-1.08) |
| Nationality (ref: non-Jordanian) |  | **-1.74 (-2.37- -1.10)***** |  | **-2.79 (-4.09- -1.49)***** |  | **-1.66 (-2.64- -0.68)***** |  | -0.99 (-2.00-0.03) |
| Livelihood loss stress, baseline |  | -0.20 (-0.90-0.51) |  | -0.01 (-1.27-1.26) |  | 0.10 (-1.09-1.29) |  | -0.55 (-1.78-0.69) |
| Livelihood loss stress, endline |  | 0.48 (-0.32-1.28) |  | 0.44 (-1.02-1.89) |  | 0.50 (-0.91-1.91) |  | 1.25 (-0.08-2.57) |
| Livelihood loss stress, follow-up |  | **3.98 (3.38-4.58)***** |  | **3.33 (2.28-4.38)***** |  | **4.19 (3.11-5.26)***** |  | **4.41 (3.27-5.54)***** |
|  |  |  |  |  |  |  |  |  |
| **Mental Health** |  | n=176 |  | n=67 |  | n=58 |  | n=51 |
| Age |  | -0.21 (-0.64-0.22) |  | -0.28 (-2.29-1.73) |  | -0.99 (-3.46-1.48) |  | -0.01 (-1.66-1.63) |
| Gender, (ref: girls) |  | -1.82 (-4.22-0.59) |  | -0.22 (-4.55-4.12) |  | -1.09 (-5.42-3.24) |  | -5.98 (-9.96- -2.01) |
| Nationality (ref: non-Jordanian) |  | **-3.05 (-5.89- -0.22)*** |  | -0.57 (-7.03-5.88) |  | -4.21 (-8.74-0.32) |  | -2.78 (-7.29-1.73) |
| Livelihood loss stress, baseline |  | 1.71 (-0.90-4.33) |  | 1.23 (-3.48-5.95) |  | -0.65 (-5.15-3.85) |  | 6.57 (1.98-11.16) |
| Livelihood loss stress, endline |  | 0.35 (-2.36-3.05) |  | 0.90 (-4.45-6.25) |  | 0.88 (-4.62-6.38) |  | 1.77 (-2.56-6.11) |
| Livelihood loss stress, follow-up |  | -0.12 (-3.03-2.80) |  | 2.79 (-2.93-8.51) |  | 1.92 (-3.81-7.64) |  | **-7.59 (-12.75- -2.44)**** |
|  |  |  |  |  |  |  |  |  |
| **Developmental Assets** |  | n=251 |  | n=117 |  | n=69 |  | n=65 |
| Age |  | 0.09 (-0.16-0.34) |  | -0.45 (-1.33-0.43) |  | -0.03 (-1.52-1.45) |  | 0.30 (-1.02-1.62) |
| Gender, (ref: girls) |  | -0.28 (-1.71-1.16) |  | 0.29 (-1.68-2.26) |  | **-2.76 (-5.51- -0.01)*** |  | 0.44 (-3.02-3.89) |
| Nationality (ref: non-Jordanian) |  | **2.55 (0.80-4.29)**** |  | **4.18 (1.28-7.09)**** |  | 2.39 (-0.52-5.30) |  | -0.16 (-3.90-3.58) |
| Livelihood loss stress, baseline |  | -0.56 (-2.19-1.06) |  | -1.82 (-3.98-0.34) |  | 1.87 (-1.39-5.12) |  | -1.06 (-5.54-3.43) |
| Livelihood loss stress, endline |  | -0.99 (-2.61-0.64) |  | -0.31 (-2.60-1.99) |  | 1.33 (-2.04-4.69) |  | **-3.76 (-7.23- -0.28)*** |
| Livelihood loss stress, follow-up |  | 0.33 (-1.44-2.09) |  | 1.39 (-1.09-3.86) |  | -2.12 (-5.63-1.38) |  | 3.60 (-1.17-8.37) |
|  |  |  |  |  |  |  |  |  |

Notes: *<0.05; **<0.01; ****P*<0.001. Caregiver protection concerns was excluded from this analysis as lost livelihood is one of the possible caregiver protection concerns and included in the outcome variable.

**Supplemental Table 3:** Primary Baseline Nationality Effect on Child Protection Concerns, Caregiver Stress, Mental Health, and Developmental Assets by Age

|  |  | **Complete Sample** |  | **Children 6-9** |  | **Children 10-12** |  | **Children 13-17** |
| --- | --- | --- | --- | --- | --- | --- | --- | --- |
|  |  | **β (95% CI)** |  | **β (95% CI)** |  | **β (95% CI)** |  | **β (95% CI)** |
|  |  |  |  |  |  |  |  |  |
| **Child Friendly Spaces Treatment** |  | n=406 |  | n=165 |  | n=114 |  | n=127 |
| Nationality (ref: non-Jordanian) |  | 0.03 (-0.06 - 0.12) |  | -0.02 (-0.16 - 0.12) |  | 0.03 (-0.14 - 0.20) |  | 0.06 (-0.11 - 0.24) |
|  |  |  |  |  |  |  |  |  |
| **Protection Concerns** |  | n=487 |  | n=235 |  | n=120 |  | n=132 |
| Nationality (ref: non-Jordanian) |  | **-2.49 (-2.99 - -1.98)***** |  | **-3.12 (-3.90 - -2.35)***** |  | **-1.77 (-2.59 - -0.96)***** |  | **-1.86 (-2.82 - -0.91)***** |
|  |  |  |  |  |  |  |  |  |
| **Caregiver Stress** |  | n=487 |  | n=235 |  | n=120 |  | n=132 |
| Nationality (ref: non-Jordanian) |  | **-1.43 (-1.80 - -1.05)***** |  | **-1.89 (-2.45 - -1.34)***** |  | **-1.04 (-1.70 - -0.37)**** |  | **-0.86 (-1.57 - -0.16)*** |
|  |  |  |  |  |  |  |  |  |
| **Mental Health** |  | n=314 |  | n=117 |  | n=93 |  | n=104 |
| Nationality (ref: non-Jordanian) |  | **-2.03 (-4.01 - -0.041)*** |  | -2.44 (-5.89 - 1.01) |  | -0.16 (-3.59 - 3.27) |  | -2.64 (-6.07 - 0.79) |
|  |  |  |  |  |  |  |  |  |
| **Developmental Assets** |  | n=463 |  | n=235 |  | n=109 |  | n=119 |
| Nationality (ref: non-Jordanian) |  | **2.57 (1.27 - 3.87)***** |  | **3.94 (2.20 - 5.69)***** |  | 0.41 (-2.23 - 3.05) |  | 1.66 (-1.04 - 4.36) |
|  |  |  |  |  |  |  |  |  |

*Notes: *<0.05; **<0.01; ***P <0.001. Bold type indicates statistical significance p<0.05.*

**Supplemental Table 4.** Baseline Participant Demographics and Descriptive Characteristics by Intervention Attendance Status

|  |  | **Entire Sample** | | |  | **CFS Attenders** | | |  | **CFS Non-attenders** | | |
| --- | --- | --- | --- | --- | --- | --- | --- | --- | --- | --- | --- | --- |
|  |  | **n = 406** | | |  | **n = 109** | | |  | **n = 297** | | |
|  |  | n/Mean |  | %/SD |  | n/Mean |  | %/SD |  | n/Mean |  | %/SD |
| **Age, T1** |  | 10.7 |  | 3.1 |  | 10.9 |  | 3.1 |  | 10.6 |  | 3.1 |
| **Gender** |  |  |  |  |  |  |  |  |  |  |  |  |
| Female |  | 220 |  | 54.2 |  | 61 |  | 56 |  | 159 |  | 53.5 |
| **Vulnerability Designation** | | |  |  |  |  |  |  |  |  |  |  |
| Yes |  | 49 |  | 12.1 |  | 11 |  | 10.1 |  | 38 |  | 12.8 |
| **Formal School Attendance** | | |  |  |  |  |  |  |  |  |  |  |
| Yes |  | 308 |  | 75.9 |  | 78 |  | 71.6 |  | 230 |  | 77.4 |
| **Extracurricular Activities** | |  |  |  |  |  |  |  |  |  |  |  |
| Always |  | 19 |  | 4.7 |  | 2 |  | 1.8 |  | 17 |  | 5.7 |
| Sometimes |  | 38 |  | 9.4 |  | 13 |  | 11.9 |  | 25 |  | 8.4 |
| Maybe |  | 348 |  | 85.9 |  | 94 |  | 86.2 |  | 254 |  | 85.8 |
| **Nationality** |  |  |  |  |  |  |  |  |  |  |  |  |
| Jordanian |  | 139 |  | 34.2 |  | 40 |  | 36.7 |  | 99 |  | 33.3 |
| Syrian |  | 241 |  | 59.4 |  | 58 |  | 53.2 |  | 183 |  | 61.6 |
| Palestinian |  | 24 |  | 5.9 |  | 12 |  | 11 |  | 12 |  | 4 |
| **Primary Caregiver in the Home** | | |  |  |  |  |  |  |  |  |  |  |
| Mother |  | 108 |  | 44.8 |  | 33 |  | 45.8 |  | 75 |  | 44.4 |
| Father |  | 125 |  | 51.9 |  | 38 |  | 52.8 |  | 87 |  | 51.5 |
| Brother |  | 5 |  | 2.1 |  | 1 |  | 1.4 |  | 4 |  | 2.4 |
| Uncle |  | 2 |  | 0.8 |  | 0 |  | 0 |  | 2 |  | 1.2 |
| Not Relative |  | 1 |  | 0.4 |  | 0 |  | 0 |  | 1 |  | 0.6 |
| **Outcome Measures, T1** | | |  |  |  |  |  |  |  |  |  |  |
| Protection Concerns |  | 3.1 |  | 2.9 |  | 3 |  | 3.1 |  | 3.1 |  | 2.9 |
| Caregiver Stress |  | 2.2 |  | 2.1 |  | 2 |  | 2.1 |  | 2.2 |  | 2.1 |
| Mental Health |  | 37.7 |  | 8.6 |  | 37.3 |  | 8.2 |  | 37.8 |  | 8.7 |
| Development Assets |  | 26.5 |  | 6.8 |  | 27.7* |  | 6.8 |  | 26* |  | 6.7 |

*Note. * Indicates a statistically significant (p<0.05) difference between groups at baseline*
