# Supplementary material for: Resilience and adjustment trajectories amongst children in displacement-affected communities in Zarqa, Jordan
Source: J Glob Health Rep. Author manuscript; Available in PMC 2022 Feb 3. (PMC8813053; doi:10.29392/001c.18233)
Supplement: 1 [file NIHMS1760452-supplement-1.pdf]

SUPPLEMENTARY MATERIALS

Online Supplementary Document

Download: https://www.joghr.org/article/18233-resilience-and-adjustment-trajectories-amongst-children-in-displacement-affected-communities-in-zarqa-jordan/attachment/48259.docx
